# Grip strength as a translational biomarker for peripheral nerve injury and recovery

**DOI:** 10.3389/fnins.2026.1916201

**Published:** 2026-07-28

**Authors:** Christoph A. Schroen

**Affiliations:** 1 Leni & Peter W. May Department of Orthopedic Surgery, Icahn School of Medicine at Mount Sinai, New York, NY, United States; 2 Medical Faculty Heidelberg, Heidelberg University, Heidelberg, Germany

**Keywords:** grasping test, grip strength, nerve injury, neuroclasis, rodent study, translational

## Abstract

A central challenge in peripheral nerve injury care is determining whether an in-continuity injury will recover spontaneously or require early surgical intervention. This determination currently depends on serial observation, during which the window for effective intervention may narrow. This Perspective discusses grip strength as a translational biomarker for peripheral nerve injury and recovery, with emphasis on rodent forelimb nerve injury models. In both rats and human patients, grip strength can be used to assess whether a motor nerve can activate its target muscle sufficiently to generate force. This shared functional endpoint gives grip strength particular translational value and distinguishes it from many preclinical functional assays used only in animal research. In rodent models of in-continuity nerve stretch injury, structurally distinct injury grades have produced markedly different grip strength recovery profiles over time, linking structural injury severity to long-term functional outcomes. Grip strength has also served as a primary functional endpoint for evaluating intraoperative electrical stimulation as an early prognostic tool and for measuring treatment effects after nerve injury. The grasping test offers a practical, reliable and low-cost method for quantifying forelimb motor recovery, but broader adoption will require standardization of testing protocols and reporting. Grip strength should not be interpreted in isolation, as it reflects the combined function of nerve signaling and target muscle force generation. Instead, it is most informative when combined with histological, electrophysiological, sensory, imaging, and clinical assessments. Standardized use of grip strength may therefore improve the translational relevance of preclinical nerve injury research.

## Introduction

Peripheral nerve injuries are often associated with great pain and severe long-term disability for patients (Bergmeister et al., 2020). In 2021 alone, over four million new cases of traumatic nerve injury occurred globally (Shi et al., 2025). Injured nerves are rarely completely disrupted and urgently repaired. More commonly, a nerve remains macroscopically intact despite an acute loss of nerve function, and the likelihood of spontaneous recovery therefore cannot be assessed in the acute clinical setting (Menorca et al., 2013). Patients may thus undergo prolonged periods of observation, even though the probability of meaningful recovery decreases as time passes without clinical improvement (Krijnen et al., 2025), and the benefit of reconstructive procedures such as nerve grafting or nerve transfer is generally greatest when intervention occurs early after injury (Beveridge et al., 2024; Lachnish et al., 2025).

This clinical uncertainty has motivated substantial preclinical work aimed at defining which structural features distinguish recoverable from non-recoverable peripheral nerve injuries (Haftek, 1970; Sinclair et al., 2012; Gluck et al., 2018; Schroen et al., 2025a). However, the translational value of structural injury classifications depends on whether they can be related to functional impairment and distinct recovery prognoses in rodents and humans alike. In rodent models, grip strength potentially offers a simple, quantitative, and repeatable measure of forelimb motor function (Lauer et al., 2022). Other commonly used outcome measures include specialized behavioral or imaging endpoints, such as sensory von Frey monofilament testing or histology and immunohistochemistry (Warner et al., 2020; Umansky et al., 2022). While these more complex assays provide detailed information on axon viability and structural integrity of the nerve, grip strength is comparatively inexpensive to measure and has a direct, clinically established human analogue in hand dynamometry (Sallam et al., 2017; Hanwright et al., 2021).

Therefore, this perspective discusses grip strength as a functional biomarker for peripheral nerve injury and recovery, with particular emphasis on its ability to relate structurally defined injury grades to motor impairment in preclinical nerve injury models. Grip strength may serve as a practical translational outcome that correlates with structural, electrophysiological, and imaging-based assessments of nerve injury in preclinical trials, potentially relating findings from preclinical rodent research to clinical evidence from studies in humans that similarly employ grip strength measurements.

## Relating injury severity to functional recovery

**FIGURE 1:**
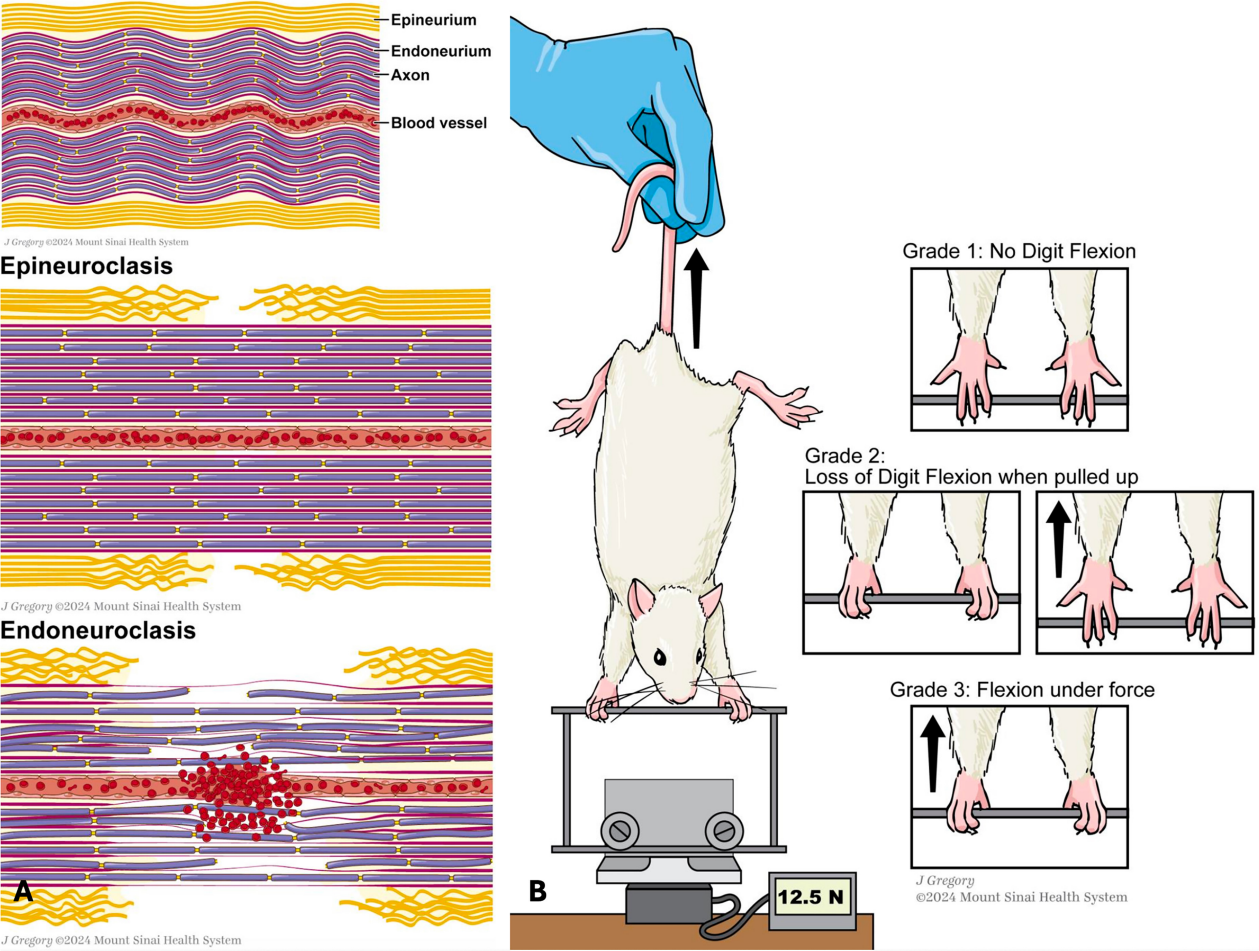
**(A)** Illustration of epineuroclasis and endoneuroclasis nerve injuries. Epineuroclasis is marked by disruption of the epineurium while the endoneurial core remains intact. Endoneuroclasis is marked by endoneurial tube disorganization and disruption of vasculature despite macroscopic continuity. Used with permission from ©Mount Sinai Health System. Originally published in: Schroen et al. (2025a). **(B)** Illustration showing the Grasping Test as a measure of grip strength in rats. Rats are held by their tail as they grab a load-cell mounted metal bar and are pulled upward until they lose grip. In addition to quantitative force measurements, the grasping test facilitates a semi-quantitative assessment of a rat’s ability to flex its digits and hold onto a metal bar while being pulled upward. Used with permission from © Mount Sinai Health System. Originally published in: Schroen et al. (2025b).

Following Wallerian degeneration, a viable axon requires intact neural connective tissues for successful regeneration. The epineurium provides a large reservoir of pro-regenerative cytokines (van Neerven et al., 2013), and endoneurial tubes are paramount to guide regenerating axons toward their target muscle (Sunderland, 1990). In-continuity nerve injuries remain a key challenge for physicians, as the internal extent of damage and thereby the likelihood of spontaneous recovery are inherently unknown (Menorca et al., 2013). Nerve injuries are commonly understood to undergo an inside-out progression of structural damage, as outlined by the Sunderland classification (Sunderland, 1951). However, a recent rodent study showed that stretch injuries follow an outside-in damage sequence with two distinct degrees of nerve injury that occur when a nerve is stretched beyond its elastic limits, called epineuroclasis and endoneuroclasis (Figure 1; Schroen et al., 2025a). Epineuroclasis is defined by disruption of the epineurium while the exposed endoneurial core remains mostly intact, whereas endoneuroclasis marks extensive axonal damage and disorganization of endoneurial tubes despite macroscopic nerve continuity (Schroen et al., 2025a,2026a). This distinction matters clinically because extensive endoneurial damage is associated with injury patterns less amenable to spontaneous recovery, yet both injury types, epineuroclasis and endoneuroclasis, are macroscopically indistinguishable during surgical exploration. The critical question is therefore whether structurally defined injury grades produce distinct and measurable functional recovery trajectories, and whether those trajectories can be captured by a single repeatable endpoint.

**FIGURE 2:**
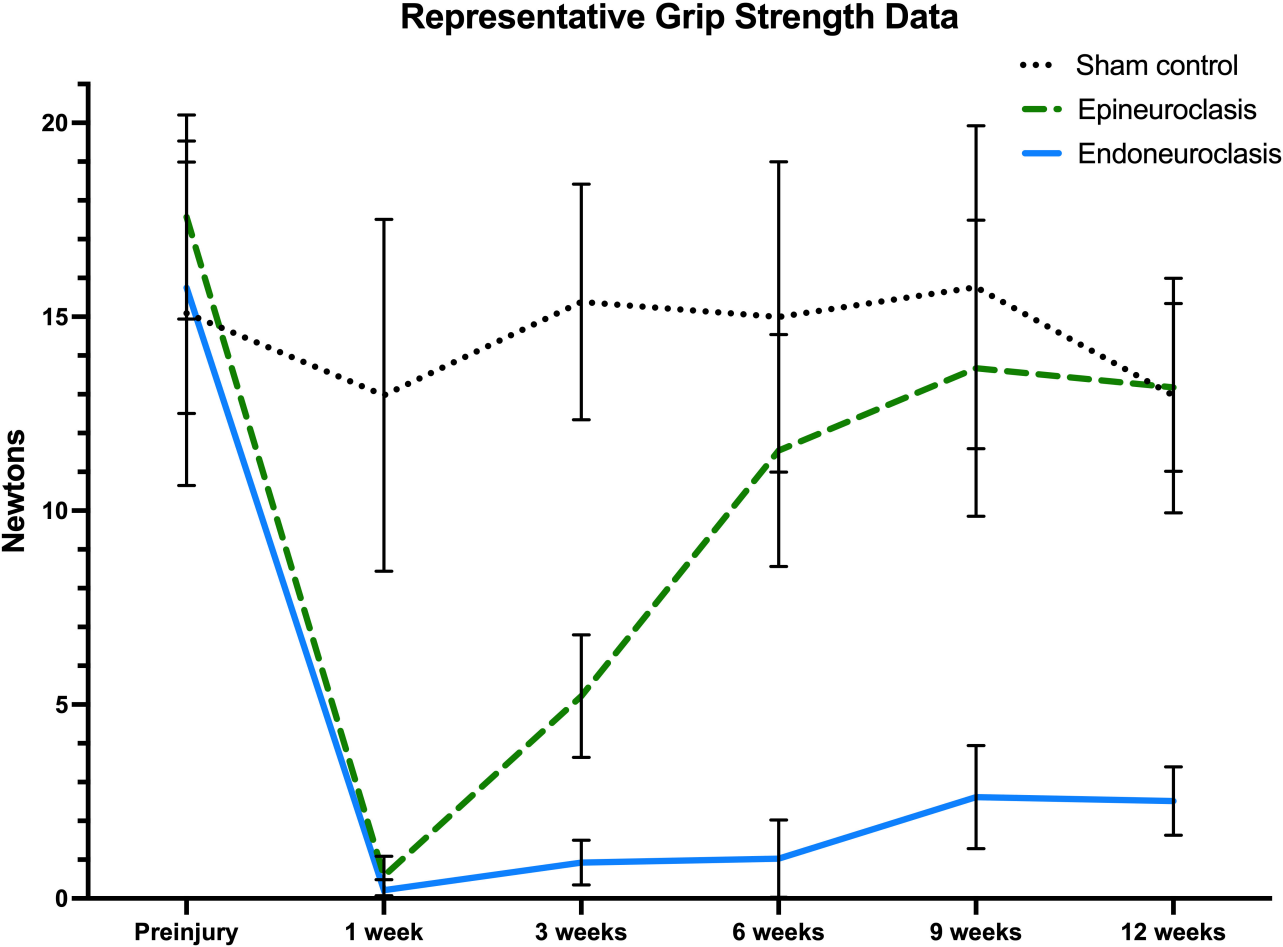
Representative grip strength data from healthy rats (*n* = 6) and rats with epineuroclasis (*n* = 8) and endoneuroclasis (*n* = 8) median nerve injuries. Grip strength test data were compared using a two-way mixed-effects model and Tukey multiple comparisons test. Both injury grades resulted in an acute loss of grip strength measured via the grasping test. However, grip strength gradually improved following epineuroclasis and reached strength similar to sham-control rats at 12 weeks, whereas endoneuroclasis injuries failed to recover at 12 weeks. This data was originally published in: Schroen et al. (2025b).

Using the grasping test to assess rat forepaw grip strength, epineuroclasis and endoneuroclasis injuries produced divergent recovery profiles (Schroen et al., 2025b). Both injury grades resulted in an acute loss of grip strength at 1 week after stretch injury. Epineuroclasis rats regained grip strength over the following weeks and reached strength values similar to uninjured sham-control rats at 12 weeks (Figure 2; Schroen et al., 2025b). Endoneuroclasis rats on the other hand failed to recover meaningful grip strength at 12 weeks, indicating a lack of functional recovery despite macroscopic nerve continuity (Schroen et al., 2025b). This study also evaluated whether an intraoperative response to electrical nerve stimulation could predict subsequent recovery and demonstrated that nerves stimulating at time zero were much more likely to recover at 12 weeks, with grip strength serving as the primary endpoint for measuring nerve function. A separate study using the same neuroclasis injury model showed that an amniochorionic membrane allograft nerve wrap accelerated recovery after epineuroclasis injury, with earlier return to baseline grip strength in treated rats compared to the untreated group (Schroen et al., 2026b). These findings highlight that grip strength measurements reveal distinct functional recovery trajectories for different grades of in-continuity nerve stretch injury in a novel rat model, which should be confirmed in larger animal models and ultimately in humans. As a behavioral endpoint, grip strength can be used to track recovery over time and distinguish injury severity during long-term follow-up. Grip strength can further be used to measure treatment effects following nerve injury in a rodent model. Both functions are essential for translational research, and both were served by a single, low-cost assay.

Other groups have measured grip strength to investigate in-continuity crush injury in the rat forelimb. Rhonchi et al. (2009) used a non-serrated clamp to apply compression to the median nerve and observed that functional recovery of grip strength started at post-injury day 12. Jager et al. (2014) used a mouse median nerve crush to induce in-continuity crush injury, where grip strength dropped to near zero by day 5, began recovering around day 10, and returned to near-preoperative levels by day 20. These crush injuries were axonotmesis injuries that correspond to a Sunderland grade 2. Multiple studies also investigated functional recovery using grip strength assays after median nerve transection and repair in rodents (Bertelli and Mira, 1995; Heinzel et al., 2020; Ritter et al., 2025). Despite the considerable differences in injury mechanism and structural damage sequence between nerve stretch, crush, and transection injuries, grip strength assays enable researchers to track recovery patterns precisely and to potentially distinguish injury type severity based on the regeneration trajectories observed. Future research should directly compare how in-continuity stretch and crush injury grades differ in their grip strength recovery trajectories.

## Measuring grip strength in rodent models

There are multiple ways to measure grip strength in animal models and specifically in rodents. Kosinsky et al. (2026) used electrical stimulation and a force transducer while rats were anesthetized to measure the maximum grip strength in the rat’s clenched paw. Munier et al. (2022) used a grip strength assay in mice that uses a microcontroller whose serial measure of resistance-based force enables the simultaneous readout of peak grip strength, the force profile (the non-linear progress of force exerted throughout a standard grip strength trial) and the cumulative force profile (the integral of force with respect to time of a single grip strength trial). Interestingly, this approach allowed the authors to identify differences in grip strength results for different types of peripheral neuropathy. Surgically induced nerve injury and statin-induced neuropathy affected grip strength and force profile, but not the cumulative force profile, whereas genetic neuropathy affected all three outcomes (Munier et al., 2022). Grip strength measurements thus enable in-depth analyses of not just peak force but also force profile, which may not only differ between different types of neuropathy but potentially also between different types and severities of traumatic nerve injury. Future research should investigate how different in-continuity injuries affect force profiles using this analysis approach. Takeshita et al. (2017) propose switching from a horizontal to a vertical configuration in mice where animals hang down from a metal bar. While the authors propose that this approach may be more valid than pulling mice horizontally, care should be taken when applying this to rats, which are generally pulled upward because falling from heights may cause serious harm due to the considerable weight of adult laboratory rats.

One grip strength assay, the grasping test, offers a highly favorable balance of validity, practicality, and translational relevance. The grasping test requires no animal training, can be completed within minutes per animal, and yields a quantitative peak force measurement (Bertelli and Mira, 1995; Papalia et al., 2003; Lauer et al., 2022). Rats are held by their tail and lowered onto a metal bar attached to a load-cell. Rats then grab the metal bar and hold onto it while the operator pulls them upward until they lose grip (Figure 1). Semi-quantitative assessment of digit flexion quality can be performed simultaneously by visually grading the animal’s ability to grab and hold onto the metal bar, providing an additional functional marker beyond raw force that may be a somewhat comparable to the Medical Research Council scale used in humans (Paternostro-Sluga et al., 2008). While not yet done, the grasping test may facilitate similarly advanced force profile analyses in rats as the mouse assay proposed by Munier et al. (2022).

Despite these advantages of the grasping test and grip strength assays in general, grip strength remains an underutilized outcome measure in peripheral nerve injury research. A main reason is that the sciatic nerve model has historically dominated preclinical nerve injury research, driven by the sciatic functional index as a well-established outcome measure (Monte-Raso et al., 2008). However, the sciatic functional index, which uses rodent footprint dimensions to compare the injured and uninjured side (de Medinaceli et al., 1982), has no direct clinical analogue and is more complex to execute and interpret. More importantly, the vast majority of peripheral nerve injuries affect the upper extremity and nerves originating from the brachial plexus (Dahlin and Wiberg, 2017). Research groups investigating peripheral nerve injuries should therefore consider using forelimb in-continuity nerve injury models and grip strength as a measure of nerve function in combination with histological evaluation and measures of sensory function. Results using this approach may hold greater translational value compared to lower extremity nerve transection and repair models using the sciatic functional index to investigate injuries that most often affect the upper extremity and frequently remain in-continuity in humans.

In the author’s experience with the grasping test in rats, using the greatest peak force of at least three consecutive trials per animal at each time point normalized to the rat’s respective pre-injury strength provides the most reliable and consistent results, as this reduces the inclusion of less-than-maximum strength values that may be caused by poor grip or habituation. Other groups propose using the mean peak force of three trials per rat and timepoint (Lauer et al., 2022). While weekly grip strength measurements may provide the greatest amount of data, measuring grip strength every second or every third week still allows researchers to reliably track recovery trends while reducing the risk of animal habituation. Broader adoption of grip strength as a preclinical measure of nerve function will require attention to standardization across studies. Variables including the number of trials per session and whether mean versus peak force is used affect the interpretability and inter-study comparability of grip strength results. Thus, establishing consistent protocols within and across laboratories would improve the validity and usefulness of grip strength as a primary outcome in preclinical nerve injury research. In rats, the author suggests using the greatest peak force of at least three consecutive trials per animal at each time point normalized to the rat’s respective pre-injury strength. The grasping test does not require animal training, but rats should be allowed to rest in the testing room for at least 15 min before testing. Sufficient time should pass between testing days to reduce the risk of animal habituation. In mice, multiple approaches and variations of the grasping test have been proposed in recent years, and future systematic reviews and comparative studies are needed to identify a protocol that may be most useful for peripheral nerve injury research.

## Grip strength as a translational biomarker in peripheral nerve injury research

A recurring limitation of preclinical nerve injury research is that the most informative outcomes provide in-depth information about a nerve’s structural integrity and conductivity, but have limited translational value for comparisons with human patients. Biopsy with histological evaluation inherently damages the nerve and is thus not an option in most human cases, while common behavioral assays such as gait analysis and the sciatic functional index lack practical and clinically useful human analogues. Grip strength on the other hand has an unusual advantage as it is a functional outcome in rodents with a direct human equivalent in hand dynamometry, and the biological question it answers is identical in rodents and humans. Both rodent and human grip strength measurements assess whether and to what extent a motor nerve can activate its target muscle sufficiently to generate grip force. Importantly, grip strength results correlate with the aforementioned, more complex outcomes. Using a rat forelimb transection model, Wang et al. (2008) found that Compound Muscle Action Potential (CMAP) amplitude strongly correlates with grip strength results, and that grip strength recovers before the CMAP amplitude displays improvements. Once CMAP improvements were detected, their correlation with grip strength results became almost linear (Wang et al., 2008). This correlation is important as Electromyography is frequently employed in human patients, supporting preclinical grip strength results as a highly translational and relevant outcome measure in nerve injury research. Ritter et al. (2025) found that CatWalk gait analysis parameters show significant alterations after median nerve transection in rats, and recovery of these parameters paralleled the return of measurable grip strength from week 6 onward on the repaired side. This study demonstrates that grip strength results clearly depict functional deficits that are similarly captured by other, often more sophisticated and less clinically translatable outcome measures. However, multiple studies demonstrate that histomorphometric analyses of injured nerves do not correlate well with grip strength results and conclude that more regenerated axons and better-looking histology do not reliably predict better grip strength (Wolthers et al., 2005; Alant et al., 2013; Lee et al., 2021). Functional measurements should therefore not replace histological evaluation of nerve injuries in preclinical research. When used in forelimb nerve injury models and combined with histological and electrophysiological evaluation, grip strength may provide one of the most clinically translatable functional outcomes in preclinical nerve injury research.

## Grip strength as a measure of nerve function in humans

Grip strength is one of the most widely used outcomes for evaluating peripheral nerve injury and function in the human upper extremity (Geere et al., 2007; Tsai et al., 2015; Bertelli et al., 2016; Buitenhuis et al., 2021). In clinical practice, grip strength is measured using handheld dynamometers and serves as an established direct measure of hand, forearm muscle and motor nerve function (Allen et al., 2020). The two most frequently employed grip strength assays are the Jamar hydraulic dynamometer, which measures force in kilograms or pounds, and the Martin Vigorimeter, which measures squeeze pressure in kilopascal (Allen et al., 2020; Bobos et al., 2020). Both have similar validity, reliability, and responsiveness, though patients tend to prefer the comfort of the Vigorimeter (Allen et al., 2020; Bobos et al., 2020). Pinch dynamometers, which measure lateral pinch, tip pinch, and palmar pinch, are used alongside grip dynamometry and are often more sensitive to specific nerve injuries (Misirlioglu et al., 2021). Pinch strength is particularly useful for evaluating ulnar nerve function, as a study by Wachter et al. (2018) showed that power grip drops by 27% while pinch strength drops by 58% after distal ulnar nerve block. In high nerve injuries, power grip is one of the three best prognostic markers for predicting functional recovery alongside sensibility and DASH score, as shown by Hundepool et al. (2015) in 61 patients with median, ulnar or combined nerve injuries. Together with the Medical Research Council scale, grip strength provides a practical measure of how effectively an electrical nerve signal reaches its target muscle and is converted into a muscle contraction in the setting of peripheral nerve injury (Akshintala et al., 2021; Padua et al., 2023). Evaluating nerve function via muscle strength measurements inherently adds overall muscle strength and function as a confounding factor, which is often raised as a major limitation of grip strength as a measure of nerve function (Lauer et al., 2022). This is particularly noteworthy as muscle atrophy is already detectable approximately 1 week after a muscle is denervated (Wiberg et al., 2015). However, this atrophy is usually a direct physiological consequence of nerve injury rather than a confounding variable. Motor nerve function depends on a signaling unit (the nerve) and a peripheral effector (the target muscle). If either one of those units fails, the goal of converting a central motor command into peripheral muscle movement will not be achieved. Following median and ulnar nerve repair in humans, Krarup et al. (2016) found that force recovered to approximately 80% of normal while the number of motor units recovered to only 20% of normal, indicating that grip strength can appear recovered due to remodeling of motor units even when electrophysiology shows substantial and persistent axon loss. Grip strength therefore does not measure nerve conduction in isolation, but evaluates whether the functional goal of motor signal conduction is accomplished, which is what matters most for patients and therefore for physicians as well. Grip strength represents a patient’s actual level of functioning and the ability to perform many tasks of daily living. It thereby ultimately reflects the practical impact that an upper extremity nerve injury may have on a patient’s quality of life. While patient-reported outcome measure (PROM) forms may capture functional impairment in more detail, grip strength has a direct preclinical analogue, which PROMs do not. Nonetheless, grip strength should always be evaluated in combination with sensory measurements and electrophysiological assessments to determine, track, and potentially locate the specific cause of a patient’s poor motor function in the context of peripheral nerve injury.

## Limitations

As with every outcome measure, grip strength has its limitations and should therefore not be used in isolation. In rodents, the grasping test requires that animals grasp the metal bar due to fear and exert maximum strength before letting go when pulled up. This is not always the case, and if the grasping test is repeated too frequently, rodents may get used to the experiment and stop using maximum force when pulled up. It is therefore recommended to conduct multiple trials per animal per timepoint and to leave sufficient time between follow-up timepoints to avoid habituation. Pappalia et al. (2003) further recommend that a single researcher should perform all grip strength experiments to reduce inter-operator variability, and Munier et al. (2022) showed that adding outcomes like force profile and cumulative force provides more granular data than peak strength alone (Lauer et al., 2022). As a measure of muscle strength, grip strength assays like the grasping test in rats do not directly evaluate nerve function itself. In preclinical nerve injury research, histological evaluation is thus important for correlating the degree of structural damage on histology with the observed functional deficit during grip strength testing. In human patients, grip strength results should be interpreted in combination with PROMs and additional studies including electrophysiological assessment and nerve imaging such as ultrasound and magnetic resonance imaging (Zaidman et al., 2013) to evaluate injury severity and ultimately the likelihood for recovery after nerve injury. Furthermore, many nerve injuries involve functions and anatomical regions that do not affect upper extremity grip strength, and grip strength is therefore only useful as a measure of nerve function when an injury occurs in a nerve that affects it. Depending on the location of a nerve lesion, muscle groups may remain partially innervated and other nerves and muscle groups may compensate for the strength lost by the injury, potentially leading to falsely reassuring grip strength results. Additionally, some nerve injuries may not affect grip strength entirely. Ulnar nerve injuries show a much more severe loss of function when intrinsic hand muscles are evaluated using the Rotterdam Intrinsic Hand Myometer instead of general grip strength measurements (Schreuders et al., 2004). Furthermore, grip strength alone does not allow physicians to evaluate a patient’s dexterity or sensory function, and force measurements require patient cooperation and active participation, which may be limited by pain. Thus, grip strength should not be used as a standalone outcome measure in preclinical research, nor as the only measure of nerve function in human patients. Grip strength should be understood as an objectively measurable functional biomarker that reflects the integrated performance of motor nerve signaling, target muscle function, pain, effort, sensory feedback, and other factors. Nonetheless, grip strength as an easy-to-measure preclinical and frequently employed clinical outcome measure holds a unique translational value and should therefore be employed more frequently in preclinical nerve injury research.

## Conclusion

A central challenge in peripheral nerve injury care is determining whether an in-continuity injury will recover spontaneously or require early surgical intervention. This determination currently depends on serial observation, during which the window for effective intervention may narrow. In both rats and human patients, grip strength is often measured to determine whether a motor nerve can activate its target muscle to generate force. This shared functional endpoint gives grip strength measurements particular translational value and distinguishes them from many preclinical functional assays that are used only in animal research. In a rodent nerve injury model, grip strength recovery profiles differed markedly between two structurally distinct grades of in-continuity injury over time. This link between structural injury severity and long-term grip strength results facilitated the preclinical validation of intraoperative electrical stimulation as a prognostic tool for acute nerve injuries at time zero rather than after months of observation. Standardized preclinical use of grip strength assays combined with histological and electrophysiological evaluation may therefore improve the translational relevance of preclinical nerve injury research and produce results more directly applicable to the clinical setting.

## Data Availability

The original contributions presented in this study are included in the article/supplementary material, further inquiries can be directed to the corresponding author.
